# Complete Genomic Sequence of *Dengue virus 1*, Isolated from Plasma Collected from a Haitian Child in 2014

**DOI:** 10.1128/genomeA.00331-17

**Published:** 2017-06-01

**Authors:** Maha Elbadry, Sarah White, Julia Loeb, Massimiliano Tagliamonte, Marco Salemi, J. Valery Madsen Beau De Rochars, Bernard Okech, Glenn Morris, John Lednicky

**Affiliations:** a Department of Environmental and Global Health, College of Public Health and Health Professions, University of Florida, Gainesville, Florida, USA; b Emerging Pathogens Institute, University of Florida, Gainesville, Florida, USA; c Department of Infectious Diseases and Pathology, College of Veterinary Medicine, University of Florida, Gainesville, Florida, USA; d Department of Pathology, Immunology and Laboratory Medicine, College of Medicine, University of Florida, Gainesville, Florida, USA; e Department of Health Services Research, Management and Policy, University of Florida, Gainesville, Florida, USA; f Department of Medicine, College of Medicine, University of Florida, Gainesville, Florida, USA

## Abstract

An outbreak of dengue fever followed a chikungunya fever outbreak in Haiti in 2014. We detected *Dengue virus 1* (DENV-1) in plasma samples collected between May 2014 and February 2015. A representative isolate was fully sequenced, and phylogenetic analyses indicate that it groups within the genotype V South American and Caribbean DENV-1 clades.

Dengue viruses are single-stranded, positive-sense RNA viruses of the genus *Flavivirus*, family *Flaviviridae*. There are five closely related serotypes (1 to 5) (1) that cause dengue fever (DF) in humans. Dengue viruses are transmitted by mosquitoes, and infections by the viruses are often asymptomatic. In about 20% of those infected, DF occurs, wherein patients present with high fever, severe frontal headache, arthralgia, and a widespread rash. In a few cases, DF progresses to a severe condition termed dengue hemorrhagic fever, which can result in a life-threatening condition called dengue shock syndrome (2). Despite being listed by the World Health Organization as a neglected tropical disease, DF is currently endemic in more than 100 countries, with an estimated 390 million infections annually (3). Economic losses in the Americas due to DF can reach USD2.1 billion per year (4). Despite its long presence, little is known about the etiology, epidemiology, or history of DF in Haiti.

We are studying arbovirus infections in Haiti and detected dengue virus infections in children during and following a chikungunya fever (CF) outbreak in 2014. The patient population consisted of children from a school clinic in Gressier, Ouest Department, Haiti, who presented with undifferentiated fevers. It had initially been assumed by clinicians that another CF outbreak had started. Instead, *Dengue virus 1* (DENV-1) viral genomic RNA (vRNA) was detected by RT-PCR (5) in a few of the plasma samples collected from the children between May 2014 and February 2015. Other viruses, such as *Zika virus* (ZIKV), were also detected (6). As our experience has been that RT-PCR tests may yield false-negative results due to many factors, the plasma samples were also inoculated onto a variety of cell lines, resulting in significant detection of more true positives. The complete genome sequence of one of the isolates from MRC-5 cells was obtained using Sanger sequencing and a genome-walking approach similar to what we have used for ZIKV (6). Briefly, cDNA was generated using reverse primers and Accuscript High Fidelity reverse transcriptase (Agilent Technologies, Santa Clara, CA, USA), and PCR using Phusion polymerase (New England Biolabs) and specific primers to produce overlapping amplicons. The 5′ and 3′ ends of the viral genome were amplified for sequencing using a rapid amplification of the cDNA Ends (RACE) kit (Life Technologies, Inc., Carlsbad, CA, USA).

DENV-1 has five different lineages (genotypes I to V) (7, 8). The genome sequence of the DENV-1 isolate from Haiti belongs to genotype V and shares a 99% nucleotide identity with the only available full-genome virus isolate obtained from a traveler from Haiti in 2010 (GenBank accession no. Ku509264.1). Phylogenetic analyses indicate that it groups within the South American and Caribbean DENV-1 clades. With this isolate fully sequenced, we now have a new standard for comparison, and this will help our studies on the introduction and dynamics of DENV-1 transmission and endemicity in Haiti.

## Accession number(s). 

The completed DENV-1 sequence of this study has been deposited in the GenBank database under the accession number KT279761.2. 
